# Supplementary material for: Screening of Staphylococcus aureus for Disinfection Evaluation and Transcriptome Analysis of High Tolerance to Chlorine-Containing Disinfectants
Source: Microorganisms. 2023 Feb 14;11(2):475. doi: 10.3390/microorganisms11020475 (PMC9967416; doi:10.3390/microorganisms11020475)
Supplement: Supplementary file 1 [file microorganisms-11-00475-s001.zip › microorganisms-2200272-supplementary.pdf]

Table S1. Functional classification of 14 strains drug resistance factors predicted by CARD

ARO Term	AMR Gene Family	Drug Class	Resistance Mechanism
LmrS	major facilitator superfamily (MFS) antibiotic efflux pump	macrolide antibiotic, aminoglycoside antibiotic, oxazolidinone antibiotic, diaminopyrimidine antibiotic, phenicol antibiotic	antibiotic efflux
mgrA	ATP-binding cassette (ABC) antibiotic efflux pump, major facilitator superfamily (MFS) antibiotic efflux pump	fluoroquinolone antibiotic, cephalosporin, penam, tetracycline antibiotic, peptide antibiotic, disinfectiong agents and antiseptics	antibiotic efflux
ErmA	Erm 23S ribosomal RNA methyltransferase	macrolide antibiotic, lincosamide antibiotic, streptogramin antibiotic, streptogramin A antibiotic, streptogramin B antibiotic	antibiotic target alteration
ErmB	Erm 23S ribosomal RNA methyltransferase	macrolide antibiotic, lincosamide antibiotic, streptogramin antibiotic, streptogramin A antibiotic, streptogramin B antibiotic	antibiotic target alteration
ErmC	Erm 23S ribosomal RNA methyltransferase	macrolide antibiotic, lincosamide antibiotic, streptogramin antibiotic, streptogramin A antibiotic, streptogramin B antibiotic	antibiotic target alteration
mepR	multidrug and toxic compound extrusion (MATE) transporter	glycylcycline, tetracycline antibiotic	antibiotic efflux
mepA	multidrug and toxic compound extrusion (MATE) transporter	glycylcycline, tetracycline antibiotic	antibiotic efflux
tet(K)	major facilitator superfamily (MFS) antibiotic efflux pump	tetracycline antibiotic	antibiotic efflux
FosB	fosfomycin thiol transferase	fosfomycin	antibiotic inactivation
GlpT	antibiotic-resistant GlpT	fosfomycin	antibiotic target alteration
murA	antibiotic-resistant murA transferase	fosfomycin	antibiotic target alteration
mecA	methicillin resistant PBP2	penam	antibiotic target replacement
mecR1	methicillin resistant PBP2	penam	antibiotic target replacement
AAC(6′)-le-APH(2′′)-la	APH(2''), AAC(6')	aminoglycoside antibiotic	antibiotic inactivation
ANT(4′)-lb	ANT(4')	aminoglycoside antibiotic	antibiotic inactivation
ANT(9)-la	ANT(9)	aminoglycoside antibiotic	antibiotic inactivation

fusB	Target protecting FusB-type protein conferring resistance to Fusidic acid	fusidic acid	antibiotic target protection
rpoB	rifamycin-resistant beta-subunit of RNA polymerase(rpoB)	rifamycin antibiotic	antibiotic target alteration, antibiotic target replacement
norA	major facilitator superfamily (MFS) antibiotic efflux pump	fluoroquinolone antibiotic	antibiotic efflux
parC	fluoroquinolone resistant parC	fluoroquinolone antibiotic	antibiotic target alteration
parE	fluoroquinolone resistant parE	fluoroquinolone antibiotic	antibiotic target alteration
gyrA	fluoroquinolone resistant gyrA	fluoroquinolone antibiotic	antibiotic target alteration
arlR	major facilitator superfamily (MFS) antibiotic efflux pump	fluoroquinolone antibiotic, disinfectiong agents and antiseptics	antibiotic efflux
arlS	major facilitator superfamily (MFS) antibiotic efflux pump	fluoroquinolone antibiotic, disinfectiong agents and antiseptics	antibiotic efflux
sdrM	major facilitator superfamily (MFS) antibiotic efflux pump	fluoroquinolone antibiotic, disinfectiong agents and antiseptics	antibiotic efflux
norC	major facilitator superfamily (MFS) antibiotic efflux pump	fluoroquinolone antibiotic, disinfectiong agents and antiseptics	antibiotic efflux

Table S2. Functional classification of 13 *Staphylococcus aureus* strains virulence factors predicted by VFDB

VF class	Virulence factors	Related genes
Adherence	Cell wall associated fibronectin binding protein	*ebh*
	Clumping factor A	*clfA*
	Clumping factor B	*clfB*
	Collagen adhesion	*cna*
	Extracellular adherence protein/MHC analogous protein	*eap/map*
	Intercellular adhesin	*icaD*
	Ser-Asp rich fibrinogen-binding proteins	*sdrC*
		sdrD
Enzyme	Serine protease	*splA*
		splB
		splC
		splD
		splE
		splF
Immune evasion	AdsA	*adsA*
	CHIPS	*chp*
	SCIN	*scn*
Secretion system	Type VII secretion system	*esaB*
		esaD
		esaE
		esxB
		esxC
		esxD
Toxin	Enterotoxin A	*sea*
	Enterotoxin B	*seb*
	Enterotoxin0like K	*selk*
	Enterotoxin0like Q	*selq*
	Exotoxin	*set*
	Leukotoxin D	*lukD*
